# Acute fulminant necrotizing amebic colitis in a pediatric patient: a rare complication of amebiasis with high mortality—a case report

**DOI:** 10.1186/s43159-020-00039-7

**Published:** 2020-09-17

**Authors:** Samantha Kiriwaththuduwa, Romola Gnanapragasam, Anjalie Amarasinghe, Yugantha Adikari, Shanika Ranasinghe, Rumala Morel, Chanuka Dharmaratne, Lakmalee Bandara

**Affiliations:** 1 Pediatric Surgery, Sirimavo Bandaranayake Specialized Children’s Hospital, Peradeniya, Sri Lanka; 2 Anesthesia/Surgical Intensive Care Unit, Sirimavo Bandaranayake Specialized Children’s Hospital, Peradeniya, Sri Lanka; 3 Department of Parasitology, Faculty of Medicine, University of Peradeniya, Peradeniya, Sri Lanka

**Keywords:** Acute fulminant necrotizing amebic colitis, *Entameba histolytica*, Case report, Child, Sri Lanka

## Abstract

**Background:**

The majority of amebic infections among humans remain asymptomatic. Rarely, the disease takes a fulminant acute course due to the development of necrotizing amebic colitis. This complication is usually found in adult patients. However, on the contrary, this case was diagnosed in a 9-year-old patient. He was transferred to the Sirimavo Bandaranayake Specialized Children’s Hospital (SBSCH), Peradeniya from the District General Hospital, Kilinochchi. To our knowledge, this is the first report of this rare complication in a child in Sri Lanka.

**Case presentation:**

We present a case of acute fulminant necrotizing amebic colitis in a 9-year-old boy. Surgical exploration revealed extensive ulceration and multiple perforations in the entire colon. PAS-Martius Yellow 40 stain highlighted amebae with erythrophagocytosis within the necrotic debris of the ulcers. The polymerase chain reaction (PCR) that was conducted to confirm the diagnosis was positive for *Entameba histolytica.* The post-operative course was marked with antimicrobial treatment for septicemia and the need for ventilator assistance. Antimicrobial treatment included intravenous metronidazole. The patient progressively recovered and was discharged on a normal diet.

**Conclusion:**

This case reports an acute fulminant necrotizing amebic colitis in a 9-year-old patient. After the treatments, the patient progressively recovered and was discharged on a normal diet. *E. histolytica* infections in northern Sri Lanka should be given attention as a public health concern. Furthermore, this case highlights that acute fulminant amebic colitis requires early surgical intervention, aggressive supportive and anti-amebic treatments. Clinicians should be cognizant of this potentially fatal complication of amebic colitis.

## Background

Amebiasis is a parasitic infection caused by *Entameba histolytica*. This infection shows a global distribution [1]. However, it is more common among populations living in congested localities with poor sanitation [2]. The majority of amebic infections among humans remain asymptomatic. Rarely, amebic infections can cause necrotizing amebic colitis. This condition has a very high mortality rate ranging from 55 to 100% if diagnosis and treatment are delayed [3]. This complication is frequently misdiagnosed as inflammatory bowel disease, and it may lead to the initiation of potentially dangerous treatment with corticosteroids [4]. Herein, we report a 9-year-old male patient presented with fulminant necrotizing amebic colitis and he recovered after total colectomy with an ileostomy, and antimicrobial treatment. This is the second case that reports this rare complication in Sri Lanka and the first case reported from a pediatric patient in the country.

## Case presentation

A 9-year-old boy from the district of Kilinochchi, in the Northern Province of Sri Lanka, was transferred to the Sirimavo Bandaranayake Specialized Children’s Hospital (SBSCH), Peradeniya from the District General Hospital, Kilinochchi on 4 January 2020. This child was visually impaired due to bilateral glaucoma. After admission to the SBSCH, the patient’s mother gave a history of fever with diarrhea, which progressed from watery to bloody diarrhea 1 week prior to the transfer.

This patient was misdiagnosed at the District General Hospital, Kilinochchi and treated for paralytic ileus associated with electrolyte imbalance for 3 days, and he was on intravenous cefotaxime.

**Fig. 1:**
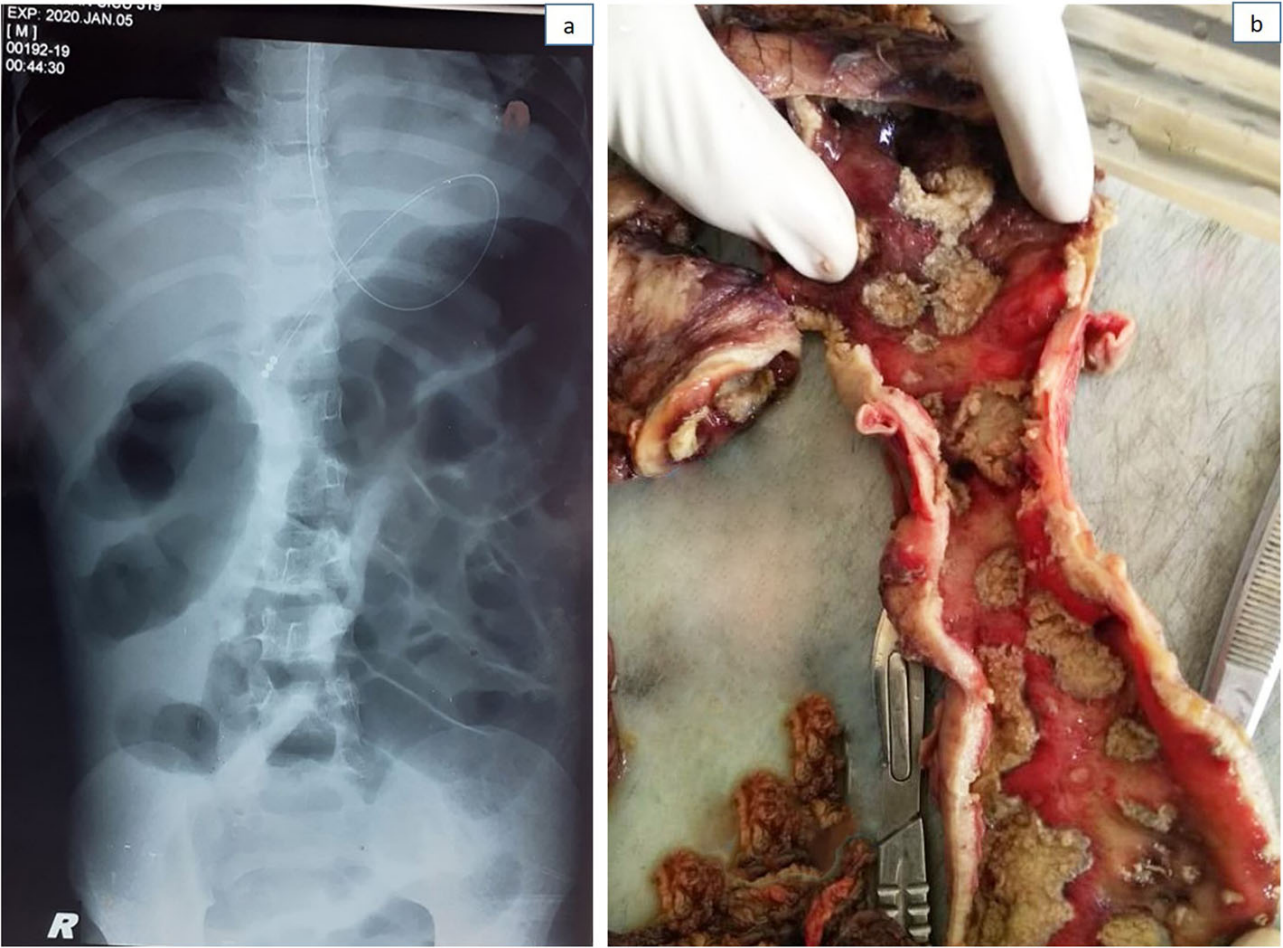
**a** X-ray image of the patient’s abdomen. The dilated bowel loops can be seen. **b** Section of the colon showing widespread amebic ulcers

On admission to the surgical intensive care unit of the SBSCH, the child was tachycardic, hypotensive, and hypothermic. He had a heart rate of 190/min, and his blood pressure was unrecordable. He had bloody diarrhea with abdominal distension and tenderness. The nasogastric tube in site had large volumes of a dark green bilious aspiration. Initial blood investigations revealed elevated inflammatory markers indicative of severe sepsis. The X-ray showed a dilated colon (Fig. 1a). The patient was resuscitated with intravenous fluid boluses and fresh frozen plasma. Noradrenalin was added as an ionotrope, and antibiotics were changed to meropenem and teicoplanin.

Surgery was performed at 6 am the next day, 8 h after he was admitted to SBSCH. Thick turbid ascetic fluid was found with multiple transmural ulcers affecting the entire colon (Fig. 1b). There were multiple concealed perforations at the hepatic and splenic flexures. The small bowel was not involved. Part of the sigmoid colon was preserved for future anastomosis. Terminal ileostomy was performed, and a mucous fistula from the distal sigmoid colon was created. Intravenous metronidazole was added post-operatively.

**Fig. 2:**
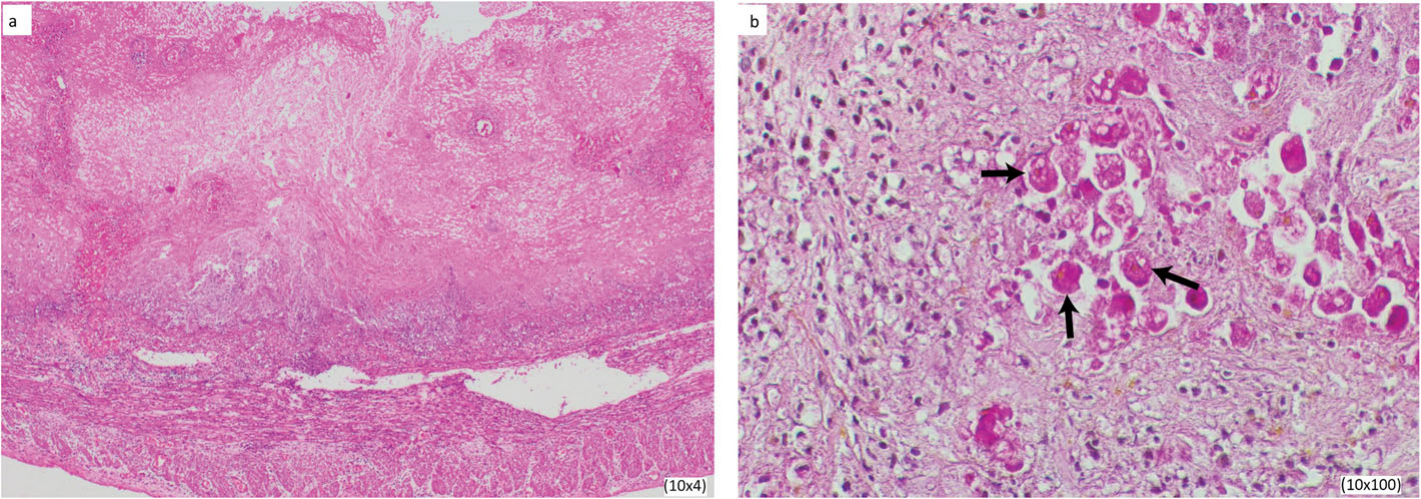
Histopathological images showing **a** typical amebic flask-shaped ulcer (10 × 4) and **b** PAS-Martius Yellow 40-stained section of the infected colonic mucosa (10 × 100). Arrows indicate the erythrophagocytic amebae

The patient initially improved but later deteriorated showing signs of ongoing peritonitis. Therefore, a re-exploration was performed to excise the remaining part of the colon as it was found to be necrotic and Hartman’s fistula was created. Specimens were sent for histological and microbiological investigations including bacterial and amebic culture. Histopathology of the colon and the caecum revealed extensive benign ulceration in colonic mucosa extending to the full thickness. Typical flask-shaped amebic ulcers were seen (Fig. 2a). Periodic Acid Schiff-Martius (PAS-Martius) Yellow 40 stain highlighted amebae with erythrophagocytosis within the necrotic debris of the ulcers (Fig. 2b). Smears and cultures were negative for amebae. The PCR that was conducted with primers specific for *E. histolytica* to confirm the diagnosis was positive for *E. histolytica* on both the ulcerated colon segments and peritoneal washings.

The post-operative course in the surgical intensive care unit was complicated by septicemia. The patient was treated with ionotropes, and ventilatory care was given for 2 weeks. Intravenous antibiotics as a combination of metronidazole, meropenem, teicoplanin, ciprofloxacin, and colestin were given. Partial parenteral nutrition was added to reduce nutritional stress as oral feeding was not possible.

The child gradually improved and 3 weeks later was moved from the surgical intensive care unit to the high dependency unit. A month after admission to the SBSCH, the patient showed good recovery and was feeding well. He was transferred to the surgical ward and subsequently discharged home on a normal diet.

## Discussion

Human infection with the protozoan *E. histolytica* causes approximately 100,000 deaths each year, and the developing world carries the main burden of morbidity and mortality [5, 6]. The main route of *E. histolytica* transmission is by ingestion of cysts from food or water contaminated with feces. The tropical climate, poor attention to hygiene and sanitation, inadequate latrine, and sanitary systems accounts for the prevalence of amebic infection in developing countries.

Fulminant amebiasis is clinically significant because of its rarity and also because the condition is difficult to diagnose and treat. Furthermore, it shows a very high mortality rate [3].The patient we are reporting was initially diagnosed as having paralytic ileus associated with bacterial dysentery. Therefore, the specific treatment for amebiasis was delayed. This led to the development of fulminant amebic colitis. This condition is often confused with idiopathic inflammatory bowel disease. Colonoscopy and colonic tissue biopsy are useful to distinguish amebiasis from other forms of colitis. Clinical symptoms, laboratory studies that include microscopic examination of feces, and X-ray findings are insufficient to make an accurate diagnosis [7]. Antigen detection in feces and serum is more sensitive and specific [8]. In this study, PAS-Martius Yellow 40 stain highlighted amebae with erythrophagocytosis within the necrotic debris of the ulcers. *E. histolytica* species-specific PCR also gave positive results. Microscopic examinations of the smears and cultures were negative. Therefore, PCR can be used to accurately diagnose fulminant amebiasis.

Development of fulminant amebic colitis is associated with various factors including the male gender and age over 60 years [9]. However, the case that we present is a 9-year-old boy. This boy’s father is a fisherman, and his mother is taking care of another 9-month-old baby. Due to this family background, his diet which normally consists of rice, fish, and lentils or a green leafy vegetable may not be supervised. Due to the lack of attention and his bilateral glaucoma which has caused severe visual impairment, he may ingest unhygienic food. Furthermore, palmyra (*Borassus flabellifer*) sap fermentation (toddy) is a common household practice in this community. This boy may have had access to toddy. Drinking toddy is known to be associated with amebiasis in the Northern Province of Sri Lanka [10].

The majority of fulminant amebic colitis cases have characteristic symptoms and signs such as severe abdominal distention and pain with peritoneal signs, sepsis with high fever, watery or bloody diarrhea, and dehydration [11, 12]. The patient in this case had bloody diarrhea with abdominal distension and tenderness.

To our knowledge, there has been one other case of acute fulminant necrotizing amebic colitis reported in Sri Lanka from a 65-year-old male patient [13]. This case was from Jaffna, Northern Province. Another study identified 346 molecular and immunologically confirmed patients with amebic liver abscess, all seen at the Teaching Hospital, Jaffna, within 3 years [14]. A more recent study identified *E. histolytica* as a public health problem in northern Sri Lanka, owing to poor sanitation and poor hygienic practices [15].

## Conclusion

In this study, we report a case of acute fulminant necrotizing amebic colitis in a 9-year-old patient. After the treatments, the patient progressively recovered and was discharged on a normal diet. *E. histolytica* infections in northern Sri Lanka should be given attention as a public health concern. Furthermore, this case highlights that acute fulminant amebic colitis requires early surgical intervention, aggressive supportive and anti-amebic treatments. Clinicians should be cognizant of this potentially fatal complication of amebic colitis.

## Data Availability

Available upon request

## Abbreviations

PCR: Polymerase chain reaction
SBSCH: Sirimavo Bandaranayake Specialized Children’s Hospital
